# Aucubin Attenuates Liver Ischemia-Reperfusion Injury by Inhibiting the HMGB1/TLR-4/NF-κB Signaling Pathway, Oxidative Stress, and Apoptosis

**DOI:** 10.3389/fphar.2020.544124

**Published:** 2020-09-08

**Authors:** Shilong Zhang, Zanjie Feng, Weidong Gao, Yuling Duan, Guoxin Fan, Xin Geng, Bo Wu, Kai Li, Kangwei Liu, Cijun Peng

**Affiliations:** ^1^ Department of Hepatobiliary and Pancreatic Surgery, Affiliated Hospital of Zunyi Medical University, Zunyi, China; ^2^ Department of Biochemistry and Molecular Biology, Zunyi Medical University, Zunyi, China

**Keywords:** ischemia-reperfusion, liver, inflammation, oxidative stress, apoptosis, aucubin

## Abstract

Liver ischemia-reperfusion injury (IRI) is a common clinical event with high morbidity in patients undergoing complex liver surgery or having abdominal trauma. Inflammatory and oxidative stress responses are the main contributing factors in liver IRI. The iridoid glucoside aucubin (AU) has good anti-inflammatory and antioxidative effects; however, there are no relevant reports on the protective effect of glucosides on hepatic IRI. The purpose of this study was to determine whether AU pretreatment could prevent liver IRI and to explore the mechanism. Sprague–Dawley rats were randomly divided into five groups. The sham operation and IRI control groups were given intraperitoneal injections of normal saline, while the AU low-dose (AU-L) group, AU medium-dose (AU-M) group, and AU high-dose (AU-H) group were given intraperitoneal injections of AU at doses of 1, 5, and 10 mg/kg/day, respectively. After 10 d, liver IRI (70% liver ischemia for 1 h, reperfusion for 6 h) was surgically established in all groups except the sham group. Our results confirmed that liver injury was significantly aggravated after hepatic ischemia-reperfusion. AU alleviated the increase of transaminase and pathological changes induced by ischemia-reperfusion and improved liver damage. AU could also ameliorate the inflammatory and oxidative stress responses induced by ischemia-reperfusion and reduced expression of high mobility group protein (HMG)B1, receptor for advanced glycation end-products (RAGE), tumor necrosis factor (TNF)-α, interleukin (IL)-1β, and reactive oxygen species (ROS). Moreover, AU reduced ischemia-reperfusion-induced mitochondrial dysfunction and cells apoptosis, increased peroxisome proliferator-activated receptor γ coactivator (PGC)-1α and uncoupling (UCP)2 protein expression, and reduced caspase-3, cleaved caspase-3, and Cytochrome P450 proteins (CYP) expression. To determine expression levels of the Toll-like receptor (TLR)-4/nuclear factor-κB (NF-κB) pathway-related proteins *in vitro* and *in vivo*, we also measured TLR-4, myeloid differentiation factor88 (MyD88), NF-κB P65, p-P65, I-kappa-B-alpha (IκB-α), and p-IκB-α levels. The results showed that AU effectively inhibited activation of the TLR-4/NF-κB signaling pathway. In conclusion, we showed for the first time a hepatoprotective effect for AU in liver IRI, which acted by inhibiting the HMGB1/TLR-4/NF-κB signaling pathway, oxidative stress, and apoptosis. Pretreatment with AU may be a promising strategy for preventing liver IRI.

## Introduction

Liver ischemia–reperfusion injury (IRI) is a type of liver injury caused by reperfusion after ischemic injury (Jimenez-Castro et al., 2019; Taner and Heimbach, 2019). Its clinical manifestation occur after the liver regains its blood supply with patients presenting a series of deterioration phenomena, such as liver function injury, jaundice, and even multiple organ failure (Donadon et al., 2016). The pathogenesis of IRI is closely related to aseptic inflammatory response, oxidative stress level, energy metabolism disorder, and cell apoptosis and autophagy (Go et al., 2015; Li et al., 2015; Olthof et al., 2017; Kan et al., 2018; Xu et al., 2019).

High mobility group protein B1 (HMGB1) is a highly conserved nuclear protein that is widely distributed in mammalian cells. When the body is injured, nuclear HMGB1 is acetylated and transferred to the cytoplasm (Liu et al., 2011). Extracellular HMGB1 binds to Toll-like receptor (TLR)-2, TLR-4, TLR-9, and receptor for advanced glycation end-products (RAGE), which promotes the release of reactive oxygen species (ROS), cytokines such as tumor necrosis factor (TNF)-α and interleukin (IL)-1β, and other inflammatory signals, thereby activating the inflammatory response (Zhang et al., 2017). Therefore, HMGB1 has been regarded as a potential target for the treatment of inflammatory responses and infection (Andersson and Tracey, 2011).

TLRs are extensively studied transmembrane protein receptors known for being the first line of defense against pathogen invasion. They play a key role in inflammation, immune cell regulation, cell proliferation, and apoptosis (Hua and Hou, 2013). TLR-4 is the most studied TLR and plays an important role in the process of heart, brain, kidney, and liver IRI (Zhao et al., 2014; Chen et al., 2016; Kadono et al., 2017; Liu et al., 2017). Meanwhile, RAGE is a member of the immunoglobulin superfamily and acts as a cellular signal transduction receptor that binds to ligands such as AGE and HMGB1. Although RAGE does not mediate renal injury or renal IRI (Dessing et al., 2012), it does play an important role in heart, liver, lung, and limb IRI (Sharma et al., 2013; Zhao et al., 2017; Wang et al., 2018; Mi et al., 2019). In addition to TLR-4 and RAGE specifically, the TLR-4/NF-κB signaling pathway is also known to play an important role in the pathogenesis of liver IRI (Zeng et al., 2009; Sherif and Al-Shaalan, 2018).

**Figure 1:**
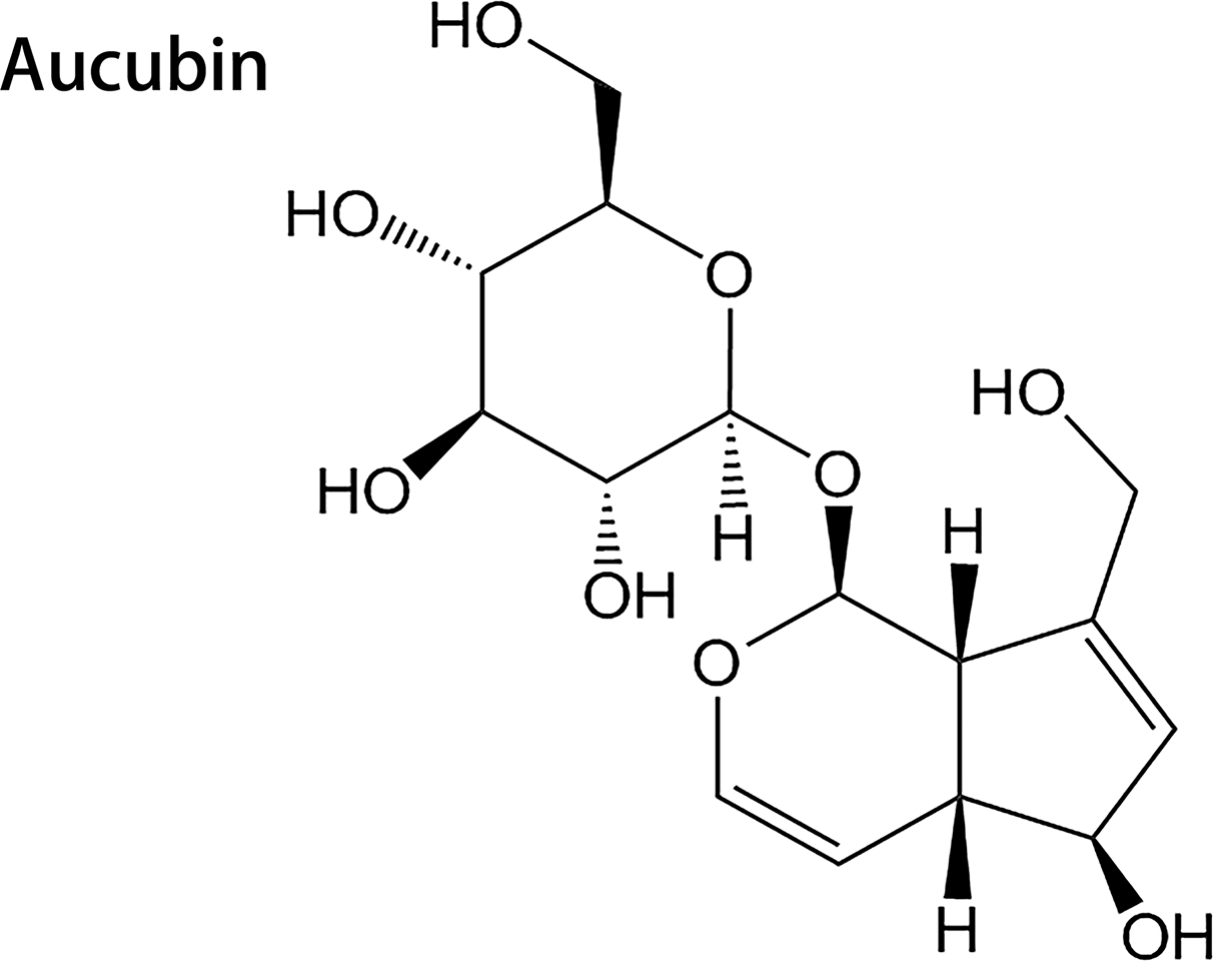
Chemical structural formula of aucubin.

Drugs and ischemic pretreatment methods are commonly used in clinical practice in effort to prevent liver IRI; however, there have not been any significant breakthroughs regarding research on drug pretreatment to protect against liver IRI. One approach to identifying potential therapeutic agents is to consider substances used in traditional Chinese medicine. For instance, the main active ingredient of traditional Chinese medicines *Eucommia ulmoides*, *Rehmannia glutinosa*, and plantain is the iridoid glucoside aucubin (AU), whose chemical name is β-d-glucopyranoside (**Figure 1**). AU provides moist heat, analgesic, antihypertensive, liver protection, and antitumor effects (Park, 2013; Ma et al., 2019). In the carbon-tetrachloride-mediated acute liver injury model (Chang et al., 1983) and nonalcoholic fatty liver model (Shen et al., 2019), AU improves liver injury through its anti-inflammatory and antioxidative effects. Recent studies in mice have indicated that AU is able to improve cardiac dysfunction mediated by ischemia through its anti-inflammatory, antioxidative, and antiapoptotic effects (Duan et al., 2019). However, potential protective effects of AU in liver IRI have not yet been reported. Therefore, the purpose of the current research was to evaluate the efficacy of pretreatment with AU to reduce the extent of liver IRI and to determine whether inhibition of the HMGB1/TLR-4/NF-κB signaling pathway and reduction of oxidative stress levels and apoptosis may play a protective role in preventing liver IRI.

## Materials and Methods

### Experimental Animals

A single batch of specific pathogen-free male Sprague–Dawley rats with a body weight of 200 ± 20 g were provided by the Experimental Animal Center of Zunyi Medical University and housed at the center under a controlled environment of 18–20°C with a 12-h light/dark cycle and free access to standard chow and tap water. The animals were handled according to the Guidelines for the Operation and Management of Laboratory Animal Facilities and all protocols were approved by the Medical Ethics Committee of Zunyi Medical University (approval no: ZMUER2016-2-054).

### Experimental Design

#### Animal Experiments

Forty Sprague–Dawley rats were randomly divided into 5 groups of 8 rats each. The sham operation group and control IRI group received intraperitoneal injections of normal saline while the AU low-dose (AU-L), AU medium-dose (AU-M), and AU high-dose (AU-H) groups were treated with intraperitoneal injections of AU (purity ≥ 98%; Solarbio, Beijing, China) at doses of 1, 5, and 10 mg/kg/day (Wang et al., 2017), respectively. After 10 consecutive days of pretreatment, the rats were subjected to liver IRI as detailed below.

#### Cell Experiments

The normal human hepatocyte line HL7702 (LO2) was purchased from the cell bank of Chinese Academy of Sciences (Shanghai, China). LO2 cells were divided into a normal cell (NC) group, empty plasmid transfection (EPT) group, TLR-4 over-expression (OE) group, TLR-4 overexpression + AU (AU) group, and TLR-4 over-expression + TLR-receptor inhibitor (TAK) group. The TLR-receptor inhibitor used was TAK-242 (purity ≥ 99.95%), which was purchased from MedChemExpress (Shanghai, China). Cells of the different groups were seeded into six-well plates and RPMI-1640 medium containing 10% fetal bovine serum, 100 g/ml penicillin, and 100 g/ml streptomycin was added. The cells were cultured at 37°C in an incubator containing 5% CO_2_ for 48 h.

### Cell Transfections

LO2 cells were cultured in six-well plates as described for the cell experiments. The supplemented RPMI-1640 medium was changed when the cells reached 50% confluence. The cells were then transfected for 48 h with TLR-4 overexpression plasmid (NMID: NM_138554.1) or empty lentivirus plasmid by (multiplicity of infection; MOI = 10). Polybrene was used to improve transfection efficiency (4 μg/ml). Expression efficiency of green fluorescent protein (GFP) was observed using a fluorescence microscope.

### Liver IRI Model

**Figure 2:**
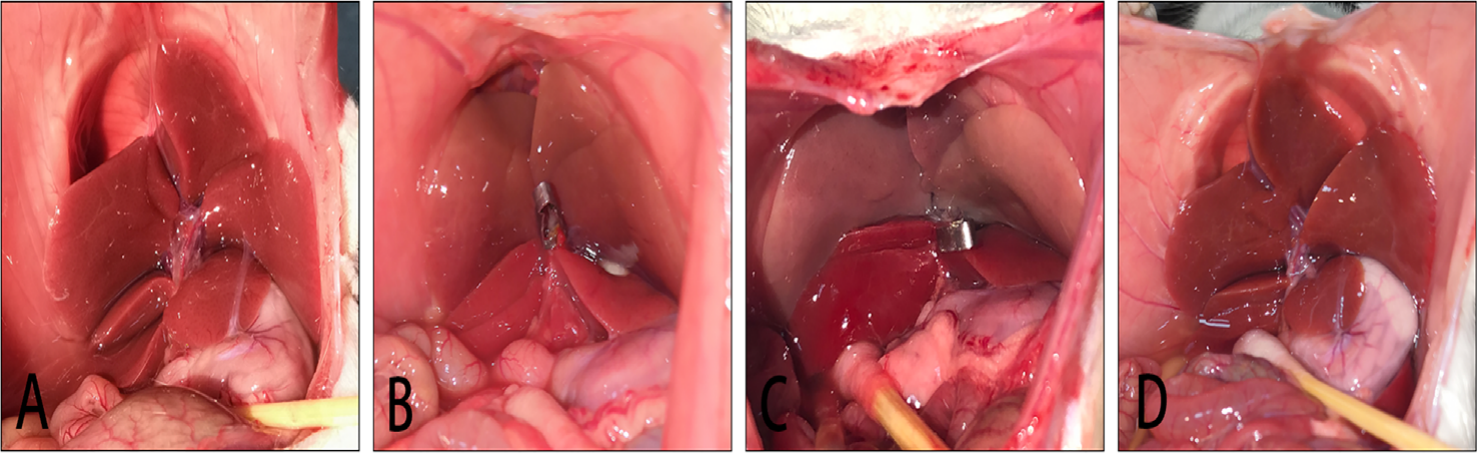
Model of liver IRI. **(A)**: liver is not ischemic; **(B)**: liver ischemia; **(C)**: after 1 h of liver ischemia; **(D)**; after 6 h of liver reperfusion.

The *in vivo* liver IRI model was established as previously described (Uto et al., 2019). Briefly, rats were anesthetized with 60 mg/kg pentobarbital (Zunyi Medical University Affiliated Hospital, Zunyi, China) (Alva et al., 2018). The anesthetized rats were placed in a supine position on the operating table and the abdominal hair removed. To disinfect the surgical area, 10% iodine and 75% ethanol were applied and a midline abdominal incision was then made. The hepatoduodenal ligament was dissected and the portal vein and hepatic artery were occluded using a vascular clamp to block blood supply to the median and left liver lobes, producing 70% liver ischemia. A change in the color of the middle and left liver lobe from bright red (**Figure 2A**) to a pale color (**Figures 2B, C**) marked success of the ischemic model. After 1 h of ischemia, the blood vessel clamp was released and a reperfusion time of 6 h was allowed. The color of ischemic liver gradually changed from dark red to bright red, which indicated success of the reperfusion (**Figure 2D**). The sham operation group underwent dissection of the hepatoduodenal ligament but liver blood-flow was not blocked. The success rate of establishing the rat HIRI model in this study was 100%.

### Collection of Blood and Liver Specimens

After 6 h of reperfusion, the rats were euthanized by cervical dislocation under anesthesia and blood and liver specimens were immediately harvested. We collected 5 ml of blood from the liver inferior vena cava and allowed it to stand at room temperature until the blood coagulated. The clotted blood was centrifuged it at 3000 rpm for 10 min and the serum collected. The serum samples were then stored at −80°C until analysis to determine levels of inflammatory factors, such as TNF-α, IL-1β, and HMGB1, and evaluation of liver function. Part of the specimens of ischemic liver tissue were fixed in a 10% neutral buffered formalin solution, and the other part was embedded in optimal cutting temperature compound (OCT) to make 10-μm thick frozen sections. The remaining fresh ischemic liver specimens were stored at −80°C until analysis.

### Liver-Tissue Homogenate Preparation

Liver tissue specimens were added to pre-cooled 0.86% of normal saline and fully ground using a SCIENTZ-48 high-flux tissue grinder (15,000 rpm, 10 s, 5 times). The ground tissue was then centrifuged at 13,000 rpm, 10 min at 4°C. The supernatant (10% liver homogenate) was collected and stored at −80°C until analysis.

### Determination of Liver Function

The separated sera were cryopreserved and transported to the laboratory of Affiliated Hospital of Zunyi Medical University. Aspartate aminotransferase (AST) and alanine aminotransferase (ALT) levels were determined using an AU5800-automatic biochemical analyzer (Beckman Coulter, California, USA).

### Liver Histopathological Examination and Scoring Criteria

The formalin-fixed liver specimens were transported to the Department of Pathology, Affiliated Hospital of Zunyi Medical University, and embedded in paraffin. Tissue sections were cut at 5-μm thickness and stained with hematoxylin and eosin (H&E). The stained sections were then examined under a light microscope. Histopathological scores ranged from 0 to 4 for each microscopic field evaluated, according to Suzuki’s liver injury criteria (Suzuki et al., 1993). Specifically, the scoring criteria were as follows: 0 = no hepatocyte injury; 1 = very few cells with hyperemia, vacuolization, or single cell necrosis; 2 = a few cells with hyperemia, vacuolization, or necrotic area < 30%; 3 = majority cell hyperemia, vacuolization, or necrosis area < 60%; and 4 = severe hemorrhage and necrotic area > 60%.

### Oxidative Stress Markers

The levels of oxidative stress markers malondialdehyde (MDA) and superoxide dismutase (SOD) were measured in the 10% liver homogenates. The amounts were determined using an MDA Kit (Beyotime, Shanghai, China) and SOD Kit (Njjcbio, Nanjing, China), according to the instructions provided by the manufacturers.

### Fluorescence Probe Techniques

Frozen liver sections were labeled for ROS superoxide anion using dihydroethidium (DHE) (Beyotime, Shanghai, China) and the nuclei were fluorescently labeled using 4′,6-diamidino-2-phenylindole (DAPI) stain (Beyotime). The sections were examined using a CellSens Dimension Imaging System (Olympus, Tokyo, Japan). Superoxide anion (red fluorescence) was observed using an excitation wavelength of 535 nm and the nuclei (blue fluorescence) observed using an excitation wavelength of 364 nm (Alexandra et al., 2020). The fluorescence area (pixel) of superoxide anion in different visual fields of the same size was counted by Image Pro Plus 6.0 software.

### Inflammatory Markers Evaluation

The expression of inflammatory factors TNF-α, IL-1β, and HMGB1 in serum was determined using commercially available enzyme-linked immunosorbent assays (ELISA; CUSABIO, Shanghai, China). Standards and experimental serum samples (100 µl) were added to wells of a 96-well plate and incubated at 37°C for 2 h. The liquid was then removed and 100-µl biotin-labeled antibody working solution was added to each well and incubate for an additional 1 h. The wells were then washed three times with washing buffer. Horseradish peroxidase-labeled avidin working solution (100 µl) was added to each well and incubated for 1 h. After removing the liquid and washing the wells five times, substrate solution (90 µl) was added and the plate incubated in the dark 37°C for 30 min. Finally, 50-µl stop solution was added to each well and the optical density (OD) values measured at 450 nm using a microplate reader.

### Real-Time Quantitative Reverse Transcription Polymerase Chain Reaaction (RT-qPCR)

HMGB1, TLR-4, and RAGE mRNA concentrations were quantitatively determined using RT-qPCR. Briefly, total RNA was isolated using RNAiso Plus reagent (TAKARA, Tokyo, Japan) and analyzed by spectrophotometer (A260/A280) to determine RNA concentrations. The RNA was reverse transcribed into complementary DNA (cDNA) and stored at −20°C until PCR analysis. For qPCR analysis, we amplified target mRNAs in the cDNA using a TB Green Premix Ex Taq II kit (TAKARA). The reactions for each of the target mRNAs consisted of 25-μl volumes including 12.5 μl TB Green Premix Ex Taq II, 1 μl specific forward primer, 1 μl specific reverse primer, 2 μl cDNA, and 8.5 μl sterile water. A standard PCR amplification procedure was performed using a two-step PCR cycle profile. Step 1 was 95°C for 30 s (1 cycle) followed by Step 2 of 95°C for 5 s and 60°C for 30 s (40 cycles). Analysis of each specimen for each target was repeated 3 times. Gene transcription was analyzed via the 2^−ΔΔCQ^ method using β-actin expression as a reference. Primer sequences of target genes are shown in **Table 1**.

**Table 1:** Primer sequences of target genes HMGB1, TLR-4, RAGE, and β-actin.

Target genes	Forward primer	Reverse primer
HMGB1	5′-ACAACACTGCTGCGGATGACAAG-3′	5′-CCTCCTCGTCTTCCTCTTCC-3′
TLR-4	5′-GCTGCCAACATCATCCAGGAAGG-3′	5′-TGATGCCAGAGCGGCTACTCAG-3′
RAGE	5′-CTGCCTCTGAACTCACAGCCAATG-3′	5′-GTGCCTCCTGGTCTCCTCCTTC-3′
β-actin	5′-CACTATCGGCAATGAGCGGTTCC-3′	5′-CAGCACTGTGTTGGCATAGAGGTC-3′

### Immunoprecipitation

Total protein was extracted using non-denatured lysate containing protease inhibitor. The protein concentration was determined using a bicinchoninic acid (BCA) protein assay kit (Solarbio). Briefly, 1 mg protein sample, 1 µg normal IgG, and 20 µl Protein A+G Agarose were combined and slowly shaken at 4°C for 2 h. The mixtures were then centrifuged at 2500 rpm, 5 min and the supernatant collected. Then, 1.44 µg HMGB1 primary antibody was added to each supernatant and incubate at 4°C overnight. The following day, 40 µl Protein A+G Agarose was added and the samples slowly shaken at 4°C for 3 h followed by centrifugation at 2500 rpm, 5 min. The supernatant was removed and the pellet washed 5 times with phosphate-buffered saline (PBS). Sodium dodecyl sulfate–polyacrylamide gel electrophoresis (SDS-PAGE) loading buffer was added to each sample and the proteins separated by electrophoresis.

### Western Blotting

Liver tissue or adherent cells were added to RIPA high-efficiency lysate buffer containing protease inhibitor and phosphatase inhibitor to extract total protein. Protein concentration was measured using the BCA protein assay kit and SDS-PAGE loading buffer was added to prepare the protein samples for electrophoresis. Appropriate amounts of protein were separated by SDS-PAGE and the protein then transferred to a polyvinylidene fluoride membrane. The membrane was blocked with 5% skimmed milk for 2 h (phosphorylated protein was blocked using 5% bovine serum albumin) and then incubated with primary antibody overnight at 4°C. The membranes were washed 3 times in tris-buffered saline and Tween 20 (TBS-T) for 5 min each. Goat anti-rabbit horseradish-peroxidase-labeled secondary antibody (1:2000; Proteintech, Wuhan, China) was added to the membranes and incubated for 1 h at room temperature. The membranes were washed 3 times with TBS-T for 10 min each. The protein bands were visualized by exposure to ultrasensitive chemiluminescence reagent ECL and using a ChemiDoc MP imaging system (Bio-Rad, Hercules, CA, USA). Image Lab version 6.0 gel imaging system software (Bio-Rad) was used to analyze the gray value of the target strip. Details on the primary antibodies, including their source and working dilutions are shown in **Table 2**.

**Table 2:** Primary antibody dilution ratio, suppliers, prefecture and countries.

Protein	Bilution ratio	Supplier	Prefecture	Country
HMGB1	1:1000	Proteintech Group	Rosemont	USA
TLR-4	1:1000	Proteintech Group	Rosemont	USA
MyD88	1:1000	Proteintech Group	Rosemont	USA
RAGE	1:1000	Proteintech Group	Rosemont	USA
IRF-1	1:1000	Proteintech Group	Rosemont	USA
P65	1:1000	Proteintech Group	Rosemont	USA
p-P65	1:1000	Affinity Biosciences	Cincinnati	USA
IκB-α	1:1000	Proteintech Group	Rosemont	USA
p-IκB-α	1:1000	Affinity Biosciences	Cincinnati	USA
TNF-α	1:1000	Bioss	Beijing	China
IL-1β	1:1000	Bioss	Beijing	China
PGC-1α	1:1000	Bioss	Beijing	China
UCP2	1:1000	Proteintech Group	Rosemont	USA
Caspase3	1:1000	Proteintech Group	Rosemont	USA
Cleaved Caspase3	1:1000	Affinity Biosciences	Cincinnati	USA
CYP-D	1:1000	Solarbio	Beijing	China
β-actin	1:4000	Affinity Biosciences	Cincinnati	USA
GAPDH	1:4000	Affinity Biosciences	Cincinnati	USA
PCNA	1:4000	Proteintech Group	Rosemont	USA

### Cell Viability Tests

To determine the appropriate concentrations of AU and TLR-receptor inhibitor TAK-242, the Cell Counting Kit-8 (CCK-8) method was used to detect cell viability and proliferation. LO2 cells were seeded into 96-well plates (3×10^3^ cells/well) and incubated overnight. The cells were then incubated for 48 h with medium containing various concentrations of AU or TAK-242. Finally, CCK-8 reagent (10 µl/well) was added and further incubated for an additional 2 h. Absorbance was measured at a wavelength of 450 nm using a microplate reader.

### Statistical Analysis

All experiments and results were performed and analyzed in a blinded manner. All data were statistically analyzed using SPSS version 23 software (International Business Machines Corporation, IBM, New York, USA). The Kolmogorov–Smirnov test was used as a distribution normality test. One-way analysis of variance (ANOVA) was used for comparison between groups. When assumed variance was equal, the least significant difference (LSD) method was used for multiple comparisons between groups. When assumed variance was not equal, the Dunnett’s T3 method was used for multiple comparisons between groups. Results are expressed as the mean ± SD. Histopathological grading did not conform to a normal distribution; therefore, nonparametric comparisons were performed using Pearson’s chi-square test. When n ≤ 40 or T ≤ 1, Fisher exact probability method was used. Significance was defined as *P* ≤ 0.05.

## Results

### AU Effects on Liver Function Tests

An automatic biochemical analyzer was used to detect serum levels of AST and ALT (**Table 3**). Compared with those of the sham operation group, serum levels of AST and ALT were significantly elevated in the AU-L, AU-M, AU-H, and IRI groups (*P* < 0.05). Compared with serum levels of AST and ALT in the IRI group, levels were significantly reduced in the AU-L, AU-M, and AU-H groups (*P* < 0.05) with the most significant reduction being in the AU-M group (*P* < 0.01).

**Table 3:** Effects of liver IR combined with intraperitoneal administration of aucubin (1, 5, and 10 mg/kg for 10 days) on serum ALT and AST levels in experimental rats.

Group	AST (U/L)	ALT (U/L)
Sham	171.00 ± 61.36	41.63 ± 13.09
IRI	1876.00 ± 114.40^α^	1243.75 ± 84.64^αβ^
AU-L	1306.38 ± 152.04^αβ^	655.50 ± 95.11^αβ^
AU-M	708.25 ± 124.39^αβ^	479.13 ± 98.30^αβ^
AU-H	1109.38 ± 144.58^αβ^	630.25 ± 105.58^αβ^

Statistical analyses were performed using the Kolmogorov–Smirnov test followed by one-way ANOVA method. All data were expressed as mean ± SD. ^α^Significant difference from the sham group at P<0.05. ^β^Significant difference from the IRI group at P<0.05.

### AU Effects on Liver Oxidative Stress Markers

Commercial MDA and SOD kits were used to measure MDA and SOD levels in liver homogenates (**Table 4**). Compared with that in the sham operation group, liver homogenate levels of MDA were significantly elevated in the AU-L, AU-M, AU-H, and IRI groups (*P* < 0.05). However, SOD levels were not significantly different in the sham operation group compared to those in the AU-M group (*P* > 0.05). The remaining three AU-treated groups had significant reductions in SOD levels (*P* < 0.05). Compared with the IRI group, liver homogenate levels of MDA were significantly reduced in the AU-L, AU-M, AU-H, and IRI groups (*P* < 0.05) and SOD levels were significantly elevated (*P* < 0.05).

**Table 4:** Effects of liver IR combined with intraperitoneal administration of aucubin (1, 5, and 10 mg/kg for 10 days) on liver homogenate MDA and SOD levels in experimental rats.

Group	MDA(µmol/g prot)	SOD(U/mg prot)
Sham	0.26 ± 0.06	90.67 ± 7.36
IRI	0.66 ± 0.06^α^	48.33 ± 4.73^α^
AU-L	0.57 ± 0.06^αβ^	62.75 ± 8.06^αβ^
AU-M	0.37 ± 0.05^αβ^	82.26 ± 9.03^β^
AU-H	0.44 ± 0.06^αβ^	72.51 ± 8.46^αβ^

Statistical analyses were performed using the Kolmogorov–Smirnov test followed by one-way ANOVA method. All data were expressed as mean ± SD. ^α^Significant difference from the sham group at P<0.05. ^β^Significant difference from the IRI group at P<0.05.

### AU Effects on Liver Inflammatory Markers

ELISA was used to detect serum levels of TNF-α, IL-1β, and HMGB1 (**Table 5**). Compared with levels in the sham operation group, serum levels of TNF-α, IL-1β, and HMGB1 were all significantly elevated in the AU-L, AU-M, AU-H, and IRI groups (*P* < 0.05). Meanwhile, serum levels of TNF-α, IL-1β, and HMGB1 were significantly reduced in the AU-L, AU-M, and AU-H groups compared with those in the IRI group (*P* < 0.05) with the most significant reduction being in the AU-M group (*P* < 0.01).

**Table 5:** Effects of liver IR combined with intraperitoneal administration of aucubin (1, 5, and 10 mg/kg for 10 days) on serum TNF-α, IL-1β, and HMGB1 levels in experimental rats.

Group	TNF-α(ng/L)	IL-1β(ng/L)	HMGB1(ng/L)
Sham	201.48 ± 17.53	51.50 ± 5.79	1058.72 ± 148.54
IRI	330.83 ± 21.36^α^	102.83 ± 10.06^α^	3864.15 ± 472.27^α^
AU-L	319.18 ± 16.36^αβ^	92.55 ± 13.73^αβ^	3120.60 ± 236.03^αβ^
AU-M	276.81 ± 18.69^αβ^	70.04 ± 7.29^αβ^	2633.70 ± 364.97^αβ^
AU-H	305.54 ± 18.93^αβ^	84.39 ± 9.19^αβ^	3077.44 ± 362.32^αβ^

Statistical analyses were performed using the Kolmogorov–Smirnov test followed by one-way ANOVA method. All data were expressed as mean ± SD. ^α^Significant difference from the sham group at P<0.05. ^β^Significant difference from the IRI group at P<0.05.

### AU Effects on Liver Histopathology

**Figure 3:**
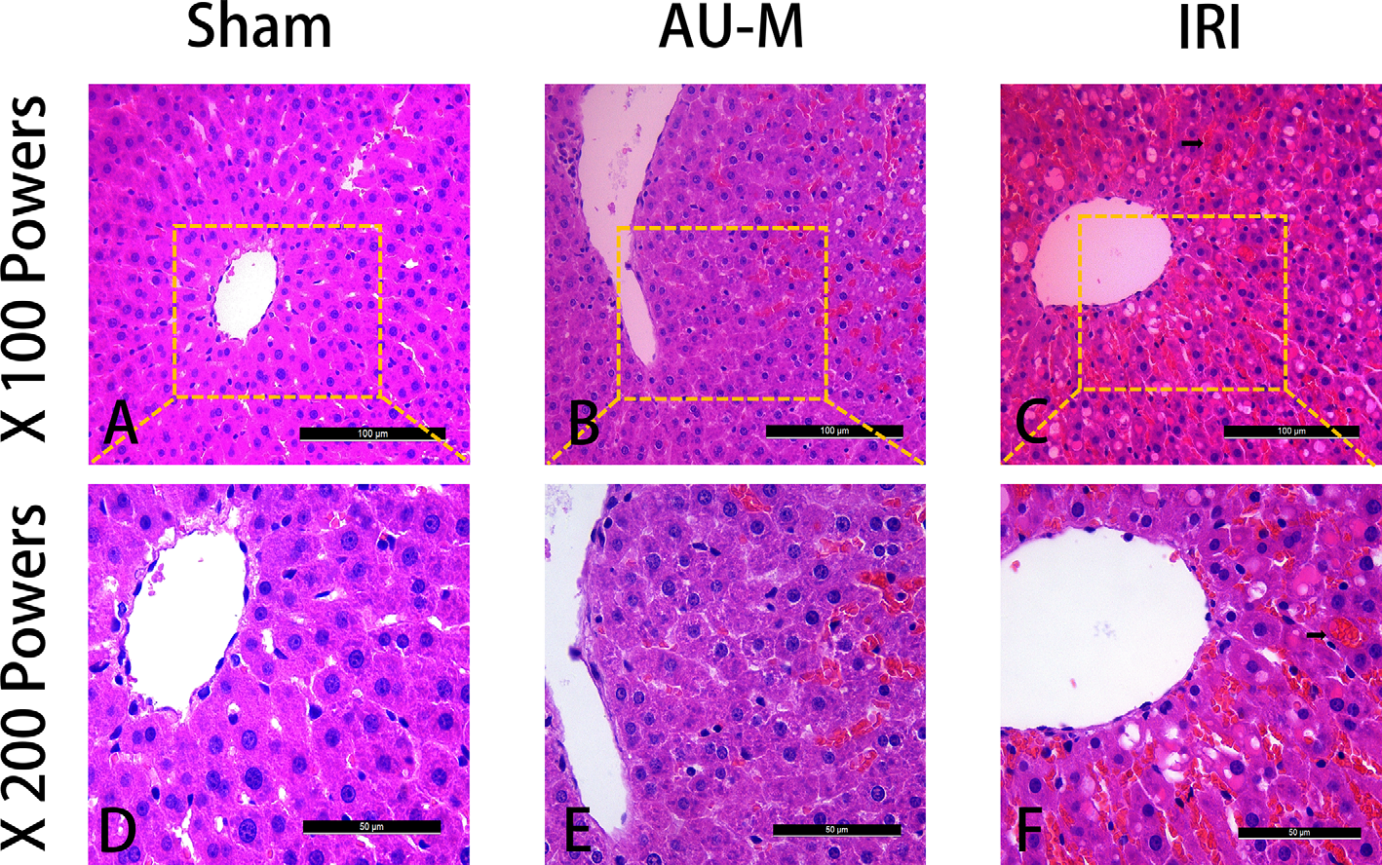
Three groups of liver sections were stained with H&E. Magnifications were low (100×) and high (200×) (scale bar=100 μm and 50 μm, respectively). The sham operation group showed normal pathology **(A, D)**. In the IRI group, the cells showed obvious degeneration and necrosis with extensive hemorrhage (arrow) **(C, F)**. The AU-M group showed mild degeneration and edema of the hepatocytes with occasional bleeding **(B, E)**.

By comparing liver function, inflammatory factors, and oxidative stress levels among the groups of rats, we found that AU (5 mg/kg) had a better protective effect on liver IRI. Thus, the sham operation, AU-M, and IRI groups were selected for follow-up experiments. H&E staining was performed on the three groups of liver tissues to evaluate histopathological changes (**Figure 3**). In the sham operation group, hepatocytes had normal morphology, arrangement, and hepatic cord structure (**Figures 3A, D**). In contrast, histopathological manifestations in the IRI group included hepatocyte edema, vacuolation, flaky necrosis, and extensive hemorrhage (**Figures 3C, F**). Meanwhile, in the AU-M group, hepatocytes showed only mild degeneration, some edema, and occasional focal hemorrhage (**Figures 3B, E**).

### AU Effects on Histopathological Score

To quantitate the differences in histopathology among the sham operation, AU-M, and IRI groups, histopathological scores were compared. Fisher’s exact test showed that the histopathological score differences among the sham, AU-M, and IRI groups were significant (*P* < 0.05; **Table 6**).

**Table 6:** Comparison of histopathological scores between sham operation, AU-M, and IRI groups.

Group	0 score	1 score	2 score	3 score	4 score	Fisher’s	*p*
Sham	7	1	0	0	0	23.415	<0.05
AU-M	2	2	3	1	0		
IRI	0	0	0	4	4		

Statistical analyses were performed using the Fisher’s exact test. Significance was predefined as P ≤ 0.05.

### AU Effects on Liver ROS

**Figure 4:**
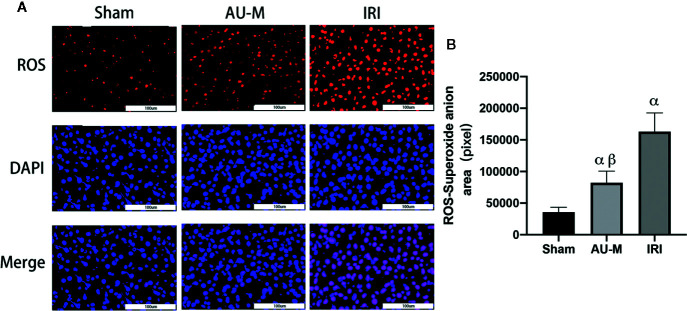
Effects of liver IR combined with intraperitoneal administration of aucubin (5 mg/kg, for 10 days) on liver ROS levels in experimental rats. Superoxide anion was expressed by red fluorescence, and nuclei by blue fluorescence; magnification was 400× and scale bar = 100 μm **(A)**. The red fluorescence area was counted by Image Pro Plus 6.0 software analysis **(B)**. Statistical analyses were performed using the Kolmogorov–Smirnov test followed by one-way ANOVA method. All data were expressed as mean ± SD. ^α^Significant difference from the sham operation group at *P* < 0.05. ^β^Significant difference from the IRI group at *P* < 0.05.

To determine ROS concentrations in the sham operation, AU-M, and IRI groups, superoxide anions and nuclei were stained using fluorescent techniques. Compared with the sham operation group, the areas of superoxide anion fluorescence in the AU-M and IRI groups were significantly increased (*P* < 0.05). Meanwhile, the area of superoxide anion fluorescence in the AU-M group was significantly decreased (*P* < 0.05) compared with that in the IRI group (**Figure 4**).

### AU Effects on Liver CYP-D

**Figure 5:**
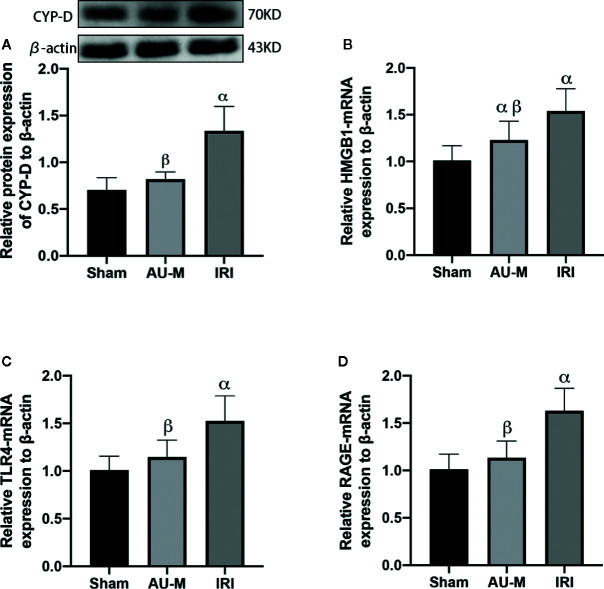
Effects of liver IR combined with intraperitoneal administration of aucubin (5 mg/kg, for 10 days) on protein expression of CYP-D **(A)** and mRNA expression of HMGB1 **(B)**, TLR-4 **(C)**, and RAGE **(D)** in the hepatic tissues of experimental rats. Statistical analyses were performed using the Kolmogorov–Smirnov test followed by One-Way ANOVA method. All data were expressed as mean ± SD. ^α^Significant difference from the sham operation group at *P* < 0.05. ^β^Significant difference from the IRI group at *P* < 0.05.

Expression levels of CYP-D in the IRI group were significantly increased compared with those in the sham operation group (*P* < 0.05). Furthermore, expression of CYP-D in the AU-M group were significantly decreased (*P* < 0.05) compared with those in the IRI group (**Figure 5A**).

### AU Effects on HMGB1, TLR-4, and RAGE mRNA Expression

Expression of HMGB1, TLR-4, and RAGE mRNA in the IRI group were each significantly increased compared with those in the sham operation group (*P* < 0.05). Furthermore, expression of HMGB1, TLR-4, and RAGE mRNA in the AU-M group were each significantly decreased (*P* < 0.05) compared with those in the IRI group (**Figures 5B–D**).

### AU Effects on the HMGB1/TLR-4/NF-κB Signaling Pathway

**Figure 6:**
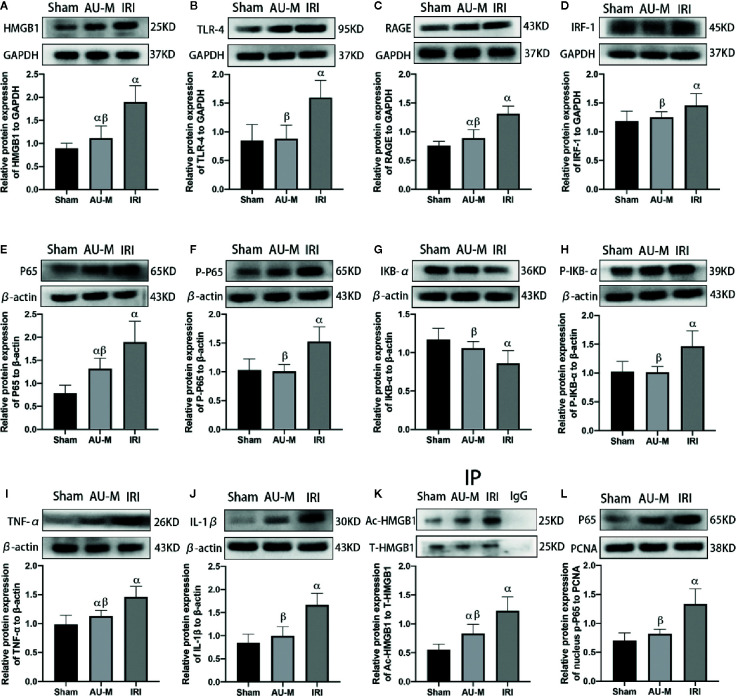
Effects of hepatic IR combined with intraperitoneal administration of aucubin (5 mg/kg, for 10 days) on quantification data and expression of HMGB1 **(A)**, TLR-4 **(B)**, RAGE **(C)**, IRF-1 **(D)**, P65 **(E)**, p-P65 **(F)**, IκB-α **(G)**, p-IκB-α **(H)**, TNF-α **(I)**, IL-1β **(J)**, acetylation HMGB1 **(K)**, and intranuclear p-P65 **(L)**, protein detected by Western blotting in the liver tissues of experimental rats. Statistical analyses were performed using the Kolmogorov–Smirnov test followed by one-way ANOVA method. All data were expressed as mean ± SD. ^α^Significant difference from the sham operation group at *P*<0.05. ^β^Significant difference from the IRI group at *P* < 0.05.

Expression levels of HMGB1, TLR-4, RAGE, interferon regulatory factor 1 (IRF-1), P65, phospho-NF-κB P65 (P-P65), phospho-I kappa B alpha (P-IκB-α), TNF-α, and IL-1β proteins in the IRI group were significantly higher than those in the sham operation group, while IκB-α expression was significantly lower (*P* < 0.05). Compared with those in the IRI group, expression levels of HMGB1, TLR-4, RAGE, IRF-1, P65, P-P65, P-IκB-α, TNF-α, and IL-1β proteins were significantly decreased in the AU-M group and protein expression of IκB-α was significantly increased (*P* < 0.05). In addition, we also found that the acetylation level of HMGB1 decreased significantly after AU pretreatment and the amount of NF-κB entering the nuclei was also significantly reduced (*P* < 0.05) (**Figure 6**).

### AU Effects on the Mitochondria-Mediated Apoptosis Pathway

**Figure 7:**
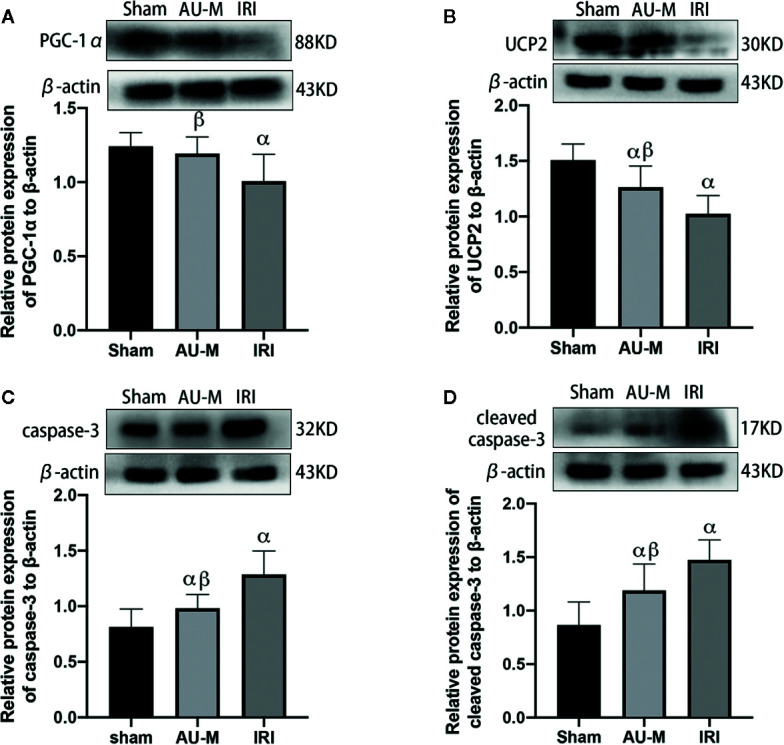
Effects of hepatic IR combined with intraperitoneal administration of aucubin (5 mg/kg, for 10 days) on quantification data and expression of PGC-1α **(A)**, UCP2 **(B)**, caspase-3 **(C)**, and cleaved caspase-3 **(D)**, protein detected by Western blotting in the liver tissues of experimental rats. Statistical analyses were performed using the Kolmogorov–Smirnov test followed by one-way ANOVA method. All data were expressed as mean ± SD. ^α^Significant difference from the sham operation group at *P* < 0.05. ^β^Significant difference from the IRI group at *P* < 0.05.

Expression levels of caspase-3 and cleaved caspase-3 proteins in the IRI group were significantly higher compared to those in the sham operation group, while PGC-1α and UCP2 protein expression was significantly lower (*P* < 0.05). Compared with those in the IRI group, expression levels of caspase-3 and cleaved caspase-3 protein were significantly decreased in the AU-M group and expression of PGC-1α and UCP2 proteins were significantly increased (*P* < 0.05) (**Figure 7**).

### Effects of AU and TAK-242 on LO2 Cell Viability

**Figure 8:**
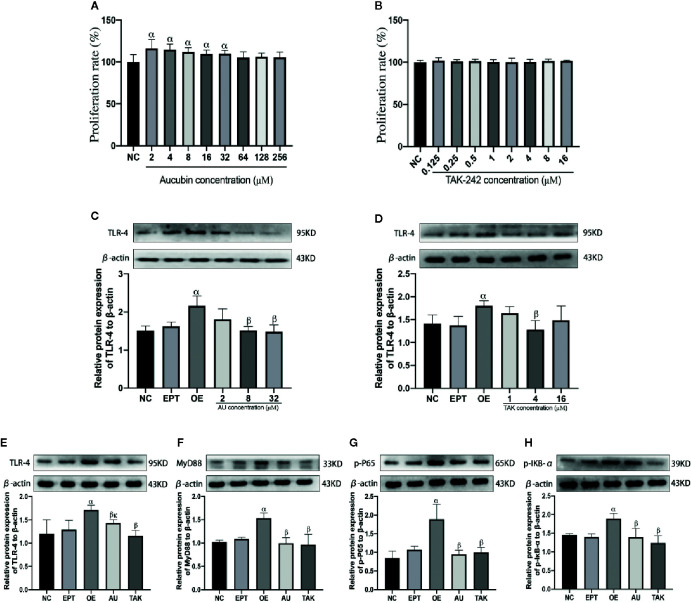
Effects of aucubin and TAK-242 on LO2 cell viability **(A**, **B)** and expression of TLR-4 **(C–E)**, MyD88 **(F)**, p-p65 **(G)**, and p-IκB-α **(H)** proteins. Statistical analyses were performed using the Kolmogorov–Smirnov test followed by One-Way ANOVA method. All data were expressed as mean ± SD. ^α^Significant difference from the NC group at *P* < 0.05. ^β^Significant difference from the OE group at *P* <0.05. ^κ^Significant difference from the TAK group at *P* < 0.05.

CCK-8 assays revealed that 2–256 µM AU had no toxic effect on LO2 cells (**Figure 8A**). Furthermore, 2–32 µM AU promoted the proliferation of LO2 cells. The TLR-receptor inhibitor TAK-242 had no toxic effect on LO2 cells at concentrations of 0.125–16 µM (**Figure 8B**).

### *In Vitro* Effects of AU on TLR-4/NF-κB Signaling Pathway Proteins

Western blotting results showed that the expression level of TLR-4 in the EPT group was not significantly changed compared with that in the NC group, but the level of TLR-4 expression in the OE group was significantly increased (*P* < 0.05). Pretreatment with AU (8 µM) and TAK-242 (4 µM) was able to inhibit protein expression of TLR-4, MyD88, p-p65, and p-I**κ**B-α (**Figures 8E–H**).

## Discussion

Liver IRI is a complex pathophysiological process involving multiple factors and is an important cause of poor prognosis and multiple organ failure in patients with complex liver surgery and liver transplantation (Pagano et al., 2018). Our current research showed that liver ischemia for 1 h followed by reperfusion for 6 h led to a significant increase in ALT and AST levels. At the same time, a series of characteristic pathological changes occurred, such as hepatocyte edema, hemorrhage, and partial hepatic parenchymal cell necrosis, which are consistent with the results of previous studies (Cai et al., 2017; Su et al., 2017). Pretreatment of rats with AU significantly improved transaminase and liver histopathological changes following liver IR. Similar protective effects were observed in a carbon-tetrachloride-induced liver injury model (Chang et al., 1983), nonalcoholic fatty liver disease model (Lee et al., 2013), and hyperlipidemia model (Shen et al., 2019). However, previous studies have not reported a protective effect of AU on IRI in important organs. The current study was the first regarding the protective effects of AU in liver IRI models.

Previous studies have demonstrated that the early stage of liver IR is characterized by oxidative-stress responses and release of ROSs that directly lead to liver cell damage (Li et al., 2016). MDA is the most important metabolite of membrane lipid peroxidation, which can aggravate cell membrane damage and alter the activity of key enzymes in the mitochondria. Meanwhile, SOD is a vitally important member of the antioxidant metalloenzymes (Xu et al., 2019). MDA, SOD, and ROS are able to directly respond to oxidative stress (Abdel-Gaber et al., 2019). AU has shown good anti-inflammatory and antioxidant effects in both acute lung injury (Qiu et al., 2018) and gastric mucosal injury (Yang et al., 2017) models. However, whether there are similar pharmacological effects in liver IRI models has not been previously studied. Our current study found that liver MDA and ROS levels were significantly increased and SOD levels were significantly decreased in the IRI group compared with those in the sham operation group, which is consistent with previous studies. Compared with that of the IRI group, AU pretreatment significantly reduced the levels of MDA and ROS, while significantly increasing the levels of SOD. This indicated that AU also had good antioxidant activity in the liver IRI model.

In addition to the oxidative stress response, the aseptic inflammation response of liver cells is also an important pathological mechanism that mediates liver IRI (Nakamura et al., 2017). The results of the current study revealed that serum levels of TNF-α, IL-1β, and HMGB1 in the IRI group were significantly higher compared to those in the sham operation group. Following pretreatment with AU, expression of TNF-α, IL-1β, and HMGB1 were each significantly lower compared to those in the IRI group, indicating that AU exhibited a good anti-inflammatory effect.

The TLR-4/NF-κB signaling pathway is a classic pathway that mediates inflammation and plays an important role in ischemic injury of the heart, brain, liver, lung, and other important organs (Sharma et al., 2013; Chen et al., 2016; Kadono et al., 2017; Liu et al., 2017; Zhao et al., 2017). During liver IR, ROS promote expression of the early response transcription factor IRF-1, enhance activity of histone acetyl transferase, and promotes acetylation of HMGB1 (Tsung et al., 2007; Doyle and Fitzpatrick, 2010; Evankovich et al., 2010; Yu et al., 2017). Acetylated HMGB1 is transferred from intracellular to extracellular environments, binds to TLR-4 and RAGE, activates the nuclear transcription factor NF-κB P65, inhibits IκB-α kinase activity, and mediates the release of inflammatory factors TNF-α and IL-1β (Xie et al., 2018). In order to determine whether AU exerts its anti-inflammatory effects through the HMGB1/TLR-4/NF-κB signaling pathway, we selected the medium dose of AU for subsequent experiments. We found that expression of HMGB1, TLR-4, and RAGE at both the mRNA and protein levels in the IRI group was significantly increased compared with that in the sham operation group. Furthermore, expression levels of NF-κB-pathway-related proteins IRF-1, P65, P-P65, and P-IκB-α were significantly increased and IκB-α protein expression was significantly reduced. Overall, these findings were consistent with previous studies (Koh et al., 2013; Qiao et al., 2014; Kim et al., 2017; Yu et al., 2017). After AU pretreatment, expression of HMGB1, TLR-4, and RAGE mRNA as well as HMGB1, TLR-4, RAGE, IRF-1, P65, P-P65, and P-IκB-α protein was significantly lower in the AU-M group compared with that in the IRI group. In contrast, expression of IκB-α protein was significantly increased. In addition, *in vitro* experiments also showed that the expression levels of TLR-4, MyD88, p-P65, and p-IκB-α proteins in LO2 cells overexpressing TLR-4 were significantly reduced following AU pretreatment. Both the *in vivo* and *in vitro* experimental results provide evidence that AU was able to reduce the inflammatory response caused by liver ischemia and reperfusion by inhibiting the HMGB1/TLR-4/NF-κB signaling pathway.

Previous studies have suggested that mitochondrial UCP2 is able to slow the process of oxidative phosphorylation, inhibit ATP production, cause cell energy metabolism disorders, and induce apoptosis (Chan et al., 2001; Stuart et al., 2001; Serviddio et al., 2008). Wan et al. (Wan et al., 2008) demonstrated the mitigation of liver IRI in obese ob/ob mice by inhibiting UCP-2 expression in fatty liver. However, the current view is that UCP2 has a double effect in the IRI model. UCP2 can also prevent ATP production and reduce the activity of mitochondria and cells and eliminate ROS produced by IRI, resulting in a protective role in liver IRI (Bi et al., 2019; Wu et al., 2019). To determine whether AU had an effect on apoptosis induced by liver IR, we measured the expression of PGC-1α, UCP2, and apoptosis execution proteins caspase-3 and cleaved caspase-3. We observed that expression of PGC-1α and UCP2 proteins was significantly reduced compared with that in the sham operation group, and levels of caspase-3 and cleaved caspase-3 were significantly increased. These findings were consistent with the previous results of Bi et al. (Le Minh et al., 2011; Bi et al., 2019). However, after AU pretreatment, PGC-1α and UCP2 expression was significantly increased and levels of caspase-3 and cleaved caspase-3 were significantly decreased. This indicated that AU was able to increase mitochondrial activity, inhibit ROS production, and reduce apoptosis by increasing PGC-1α and UCP2 protein expression, and thereby play a protective role in liver IRI.

## Conclusions

**Figure 9:**
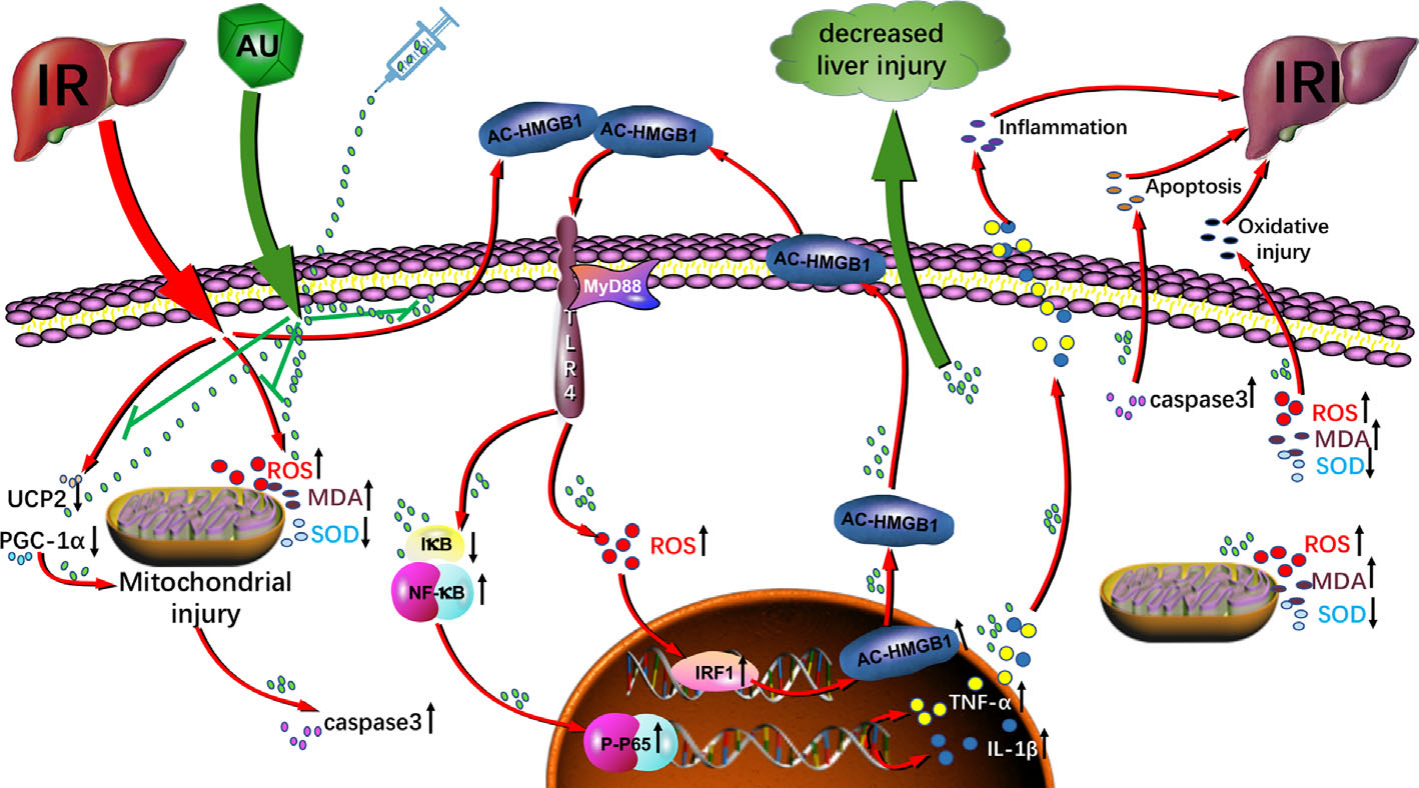
Aucubin attenuates liver ischemia-reperfusion injury by inhibiting the HMGB1/TLR-4/NF-κB signaling pathway, oxidative stress, and apoptosis. The molecular mechanism implicates downregulation of the HMGB1/TLR-4/NF-κB signaling pathway, oxidative stress level, mitochondrial dysfunction, and apoptosis.

AU decreased liver IRI by (I) reducing liver transaminase activity and liver histopathological changes; (II) inhibiting oxidative stress by reducing MDA and ROS levels and increasing SOD levels in the liver; (III) regulating the HMGB1/TLR-4/NF-κB signaling pathway to reduce expression of inflammatory factors such as HMGB1, TNF-α, and IL-1β and inhibit the inflammatory response of liver cells; and (IV) increasing mitochondrial activity that inhibits ROS generation and decreasing expression of apoptotic protein caspase-3 that inhibits apoptosis. The current study showed that AU was able to significantly reduce the degree of liver IRI through its anti-inflammatory, antioxidative, and antiapoptotic effects. The results are summarized in **Figure 9**. Although our findings provide new insight into AU and it protective activity in liver IRI, the specific mechanism through which AU regulates mitochondria injury mediated apoptosis remains unclear and further research is needed.

## Data Availability Statement

The authors acknowledge that the data presented in this study must be deposited and made publicly available in an acceptable repository, prior to publication. Frontiers cannot accept a article that does not adhere to our open data policies.

## Ethics Statement

The animal study was reviewed and approved by Medical Ethics Committee of Zunyi Medical University, with approval no: ZMUER2016-2-054.

## Author Contributions

SZ and ZF contributed in the study design, practical work, manuscript writing and revision. CP participated in research design and discussion. WG, YD, GF, XG, BW, KL, and KWL participated in the implementation of some experiments. All authors contributed to the article and approved the submitted version.

## Funding

National Natural Science Foundation of China (NO. 81660688); Science and Technology Fund Project of Guizhou Health and Family Planning Commission (no. gzwjkj2016-1-035).

## Conflict of Interest

The authors declare that the research was conducted in the absence of any commercial or financial relationships that could be construed as a potential conflict of interest.

## Abbreviations

IRI, ischemia-reperfusion injury; AU, Aucubin; AST, aspartate aminotransferase; ALT, alanine aminotransferase; MDA, malondialdehyde; HMGB1, high mobility group protein B1; TNF-α, tumor necrosis factor alpha; IL-1β, interleukin-1β; SOD, superoxide dismutase; ROS, reactive oxygen species; TLR-4, Toll-like receptors 4; RAGE, receptor for advanced glycation end-products; IRF-1, Interferon regulatory factors 1; NF-κB P65, nuclear factor kappa-B P65; P-NF-κB P65, phospho- nuclear factor kappa-B P65; IκB-α, I kappa B alpha; P-IκB-α, phospho-I kappa B alpha; PGC-1α, peroxisome proliferator activated receptor γ coactivator-1 alpha; UCP2, Uncoupling protein2; MyD88, myeloid differentiation factor88; CYP, Cytochrome P450 proteins.
